# Synthetic gRNA/Cas9 Ribonucleoprotein Inhibits HIV Reactivation and Replication

**DOI:** 10.3390/v14091902

**Published:** 2022-08-28

**Authors:** Sushant Khanal, Dechao Cao, Jinyu Zhang, Yi Zhang, Madison Schank, Xindi Dang, Lam Ngoc Thao Nguyen, Xiao Y. Wu, Yong Jiang, Shunbin Ning, Juan Zhao, Ling Wang, Mohamed El Gazzar, Jonathan P. Moorman, Zhi Q. Yao

**Affiliations:** 1 Center of Excellence in Inflammation, Infectious Disease and Immunity, Quillen College of Medicine, East Tennessee State University, Johnson City, TN 37614, USA; 2 Department of Internal Medicine, Division of Infectious, Inflammatory and Immunologic Diseases, Quillen College of Medicine, East Tennessee State University, Johnson City, TN 37614, USA; 3 HCV/HBV/HIV Program, James H. Quillen VA Medical Center, Department of Veterans Affairs, Johnson City, TN 37614, USA

**Keywords:** HIV-1, gRNA, Cas9, ribonucleoprotein, viral inhibition

## Abstract

The current antiretroviral therapy (ART) for human immunodeficiency virus (HIV) can halt viral replication but cannot eradicate HIV infection because proviral DNA integrated into the host genome remains genetically silent in reservoir cells and is replication-competent upon interruption or cessation of ART. CRISPR/Cas9-based technology is widely used to edit target genes via mutagenesis (i.e., nucleotide insertion/deletion and/or substitution) and thus can inactivate integrated proviral DNA. However, CRISPR/Cas9 delivery systems often require viral vectors, which pose safety concerns for therapeutic applications in humans. In this study, we used synthetic guide RNA (gRNA)/Cas9-ribonucleoprotein (RNP) as a non-viral formulation to develop a novel HIV gene therapy. We designed a series of gRNAs targeting different HIV genes crucial for HIV replication and tested their antiviral efficacy and cellular cytotoxicity in lymphoid and monocytic latent HIV cell lines. Compared with the scramble gRNA control, HIV-gRNA/Cas9 RNP-treated cells exhibited efficient viral suppression with no apparent cytotoxicity, as evidenced by the significant inhibition of latent HIV DNA reactivation and RNA replication. Moreover, HIV-gRNA/Cas9 RNP inhibited p24 antigen expression, suppressed infectious viral particle production, and generated specific DNA cleavages in the targeted HIV genes that are confirmed by DNA sequencing. Because of its rapid DNA cleavage, low off-target effects, low risk of insertional mutagenesis, easy production, and readiness for use in clinical application, this study provides a proof-of-concept that synthetic gRNA/Cas9 RNP drugs can be utilized as a novel therapeutic approach for HIV eradication.

## 1. Introduction

Human immunodeficiency virus (HIV) latency is historically an unresolved clinical problem, and viral eradication remains a global challenge [1,2]. While antiretroviral therapy (ART) can halt HIV replication by targeting multiple stages in the viral life cycle, ART cannot eradicate HIV infection [3,4,5]. This dilemma arises primarily because of the integration of proviral DNA into the host genome, allowing the virus to remain genetically silent in reservoir cells and be replication-competent upon the interruption or cessation of ART [6,7]. Thus, the major challenge for any HIV curative strategy is genetically eliminating the integrated proviral DNA from reservoir cells without causing collateral cytotoxic reactions [6,8]. 

There are several nuclease-based approaches (e.g., Zinc finger, Transcription activator-like effector nucleases (TALENs), homing endonucleases, recombinases, clustered regularly interspaced short palindromic repeats (CRISPR)/CRISPR-associated protein (Cas)) that are used for editing host genes necessary for HIV infection and/or proviral DNA integration into reservoir cells [6,8,9]. Recent studies have shown that CRISPR/Cas9-mediated gene-editing of incorporated proviral DNA is an appealing and feasible approach to combat latent HIV infection [9,10,11,12,13,14,15]. This approach enables high-efficient excision or mutagenesis (i.e., insertion/deletion and/or substitution) of the viral genome from anatomically privileged tissues, eliminates integrated proviral DNA, and precludes HIV reactivation [9,10,11,12,13,14,15]. The use of transient CRISPR/Cas9 treatment for HIV elimination has shown promising results in recent studies [12,16]. Despite its promise and potential for viral clearance in both in vitro and in vivo models [9,10,11], the CRISPR/Cas9 approach faces several challenges that need to be overcome before its clinical application, including off-target effects and drug delivery efficiency. Thus, the selection of HIV-targeting genes that are crucial for viral replication but avoid off-target effects, and the efficient delivery of these gene-editing drugs to HIV reservoirs, are the bottlenecks in CRISPR/Cas9-mediated HIV eradication. In addition, current delivery technologies for CRISPR/Cas9 often require viral vectors, which pose safety concerns for therapeutic application in humans [9,10,11]. These shortcomings largely limited the prospect of using the CRISPR/Cas9 approach as a therapeutic drug for HIV eradication. Notably, synthetic guide RNA (gRNA)/Cas9-ribonucleoprotein (RNP) is an attractive non-viral formulation for the CRISPR/Cas9 approach. The advantages of this approach include rapid viral DNA cleavage, low off-target effects, reduced risk of insertional mutagenesis, easy production, and readiness for use in clinical applications [17,18,19]. Since electroporation or nucleofection is not feasible for drug delivery in vivo, the development of an appropriate delivery system for gRNA/Cas9 RNP such as nanoparticles and/or exosomes is needed [20,21]. Therefore, robust pharmaceutical strategies are desperately needed for the delivery of this synthetic gRNA/Cas9 RNP drug, specifically to the reservoir cells in order to eliminate the integrated HIV DNA and achieve a sterile viral resurgence after ART cessation.

To address these unmet medical challenges, in the present study, we selected unique CRISPR targets on HIV genes that are crucial for viral replication but share no overlap (off-targeting) with the human genome. We synthesized a series of gRNA/Cas9 RNP candidates and tested their antiviral activity as well as cytotoxicity in different HIV-infected cellular models. Our results show that synthetic gRNA/Cas9 RNPs targeting HIV DNA polymerase and *vpr*/*tat* genes can efficiently inhibit HIV reactivation/replication without causing cytotoxic effects. Thus, these synthetic HIV gRNA/Cas9 RNPs warrant further investigation as a potential therapeutic drug. Meanwhile, our data highlight the importance of utilizing a new strategy based on engineered nanoparticles or exosomes for delivering potential g/RNA/Cas9 RNP drugs in vivo.

## 2. Results

### 2.1. Stimulation of Latently HIV-Infected Cells Triggers Viral Reactivation/Replication and Cell Apoptosis

**Figure 1:**
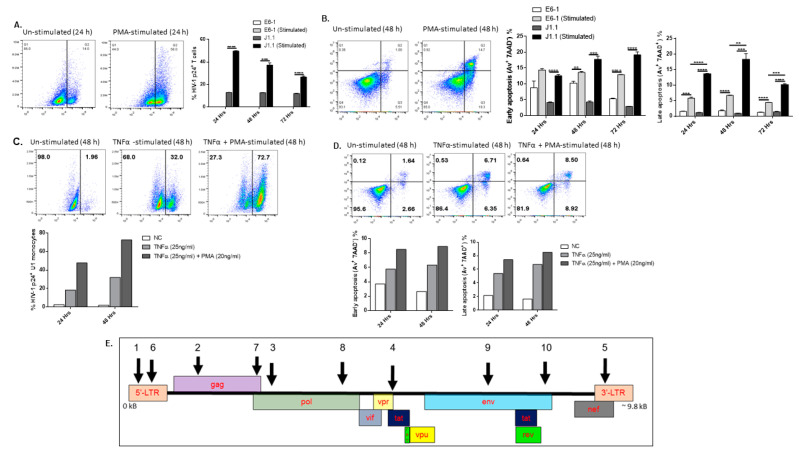
**HIV reactivation induces cellular apoptosis in J1.1 T cells and U1 monocytes.** (**A**) HIV latently infected Jurkat J1.1 and parental E6-1 cells were stimulated with 20 ng/mL PMA for 2 h, washed, and cultured for an additional 24–72 h to measure p24 expression. Representative dot plots and summary data from three independent experiments showing the percentage (%) of HIV p24 levels in E6-1 and J1.1 cells with or without PMA stimulation are shown. (**B**) Representative dot plots and summary data from three independent experiments showing the percentage (%) of early (Av^+^ 7AAD^−^) and late (Av^+^ 7AAD^+^) apoptotic E6-1 and J1.1 cells with or without PMA stimulation for 48 h. ** *p* < 0.01, *** *p* < 0.001, **** *p* < 0.0001. (**C**) Representative dot plots and summary data showing the percentage (%) of HIV p24 levels in U1 monocytes with or without TNFα and PMA stimulation for 24–48 h. (**D**) Representative dot plots and summary data showing the percentage (%) of early (Av^+^ 7AAD^−^) and late (Av^+^ 7AAD^+^) apoptotic U1 monocytes with or without TNFα and PMA stimulation for 24–48 h. (**E**) Specific gRNAs (numbered according to the order of their synthesis) targeting different sites within the HIV-1 genome (indicated by arrows) were generated using the E-CRISP online tool.

To test the efficacy and cytotoxicity of the synthesized gRNA/Cas9 RNPs, we first employed a latent HIV-harboring T cell line, J1.1 cells, which contains 1–2 copies of the replication-competent, HIV-1 proviral DNA incorporated into their genome [22,23,24]. Its parental Jurkat T cell line, E6-1, served as an HIV-negative control. Phorbol 12-myristate 13-acetate (PMA) was used for J1.1 cell stimulation/activation and HIV latency reversion per previous reports [25,26]. After determining the degrees of T cell activation and HIV replication at various time points (data not shown), we used a one-time 2 h PMA stimulation as a standard condition for the following experiments. As shown in Figure 1A, there was a low rate (~10% baseline) of p24 expression in J1.1 cells without PMA stimulation. After a one-time 2 h PMA stimulation, the number of p24^+^ cells increased significantly in stimulated cells compared with the unstimulated cells. Of note, the frequency of p24^+^ cells declined over time following the single PMA stimulation and 72 h cell culture. We have previously reported that T cell activation and HIV replication trigger unrepaired DNA damage, resulting in T cell apoptosis [27]. While PMA stimulation increased early (Av^+^ 7AAD^−^) and late (Av^+^ 7AAD^+^) apoptosis in both E6-1 (HIV-uninfected) and J1.1 (HIV-infected) cells, stimulated J1.1 T cells exhibited a significantly increased level of late apoptosis (cell death) compared with E6-1 T cells (Figure 1B), likely due to the reactivation of proviral DNA and production of pathogenic HIV particles following T cell activation by PMA stimulation.

To confirm that HIV latency reversal triggers cellular apoptosis, we also tested HIV-1 p24 and Av/7AAD expression in U1 monocytes, which harbor 1–2 copies of integrated, latent HIV-1 provirus [28,29,30]. U1 cells were stimulated with either 25 ng/mL TNFα alone or in combination with 20 ng/mL PMA for 24–48 h. The percentage (%) of HIV-1 p24^+^ cells increased in cells stimulated with TNFα plus PMA stimulation compared with TNFα alone, especially at 48 h (Figure 1C). We observed increases in both early and late apoptosis following TNFα (single) and TNF + PMA (dual stimulation), with higher levels in the dual stimulated cells at 48 h (Figure 1D). Taken together, these data suggest that cellular stimulation triggers HIV latency reactivation, viral replication, and cell apoptosis in latently HIV-infected cells.

### 2.2. Design of Virus-Specific gRNAs Targeting the HIV Genome

Selection of specific gRNAs targeting HIV proviral DNA to block viral reactivation/replication without off-targeting the human genome is the first step in developing CRISPR/Cas9-based gene-editing drugs for HIV clearance. To select the most potent targeting sites in the HIV genome, we used the E-CRISP gRNA online designer (version 5.4, provided by Dr. Boutros at the German Cancer Research Center) [31,32,33,34] to screen the HIV-1_NL4-3_ sequence (accession no. AF324493.2) for potential HIV-1 gRNA target sites. 

We selected 10 HIV-gRNAs with the best-predicted on-target (within HIV-1 sequences that are critical for viral replication) and lowest off-target (0% overlap within the human genome) values (Figure 1E). Specifically, gRNA-1 and gRNA-6 target the 5′-LTR (long terminal repeat); gRNA-2 targets the *gag* gene. Notably, gRNA-1 (5′-GCAGAACTACACACCAGGGCC-3′) and gRNA-2 (5′-GGATAGATGTAAAAGACACCA-3′) have been reported by Dash and colleagues [9]. They used a plasmid expressing gRNAs/Cas9 packaged in adenovirus, which shows a strong effect in eliminating HIV-1 in an HIV-infected humanized mouse model undergoing ART treatment. Furthermore, gRNA-7 targets the *gag*/*pol* genes; gRNA-3 and gRNA-8 target the *pol* gene; gRNA-4 targets the critical *vpr*/*tat* genes; gRNA-9 and gRNA-10 target the *env* gene; and gRNA-5 targets the 3′-LTR/*nef* sequences. According to the E-CRISP designer, gRNA-3, -4, -5, and -7 have an HIV specificity score of 100%; whereas gRNA-6, -8, -9, and -10 have a specificity of approximately 80%. All gRNAs have no (0%) overlap with the human genome. These gRNAs are designed/synthesized to target the proviral DNA sequences adjacent to the canonical NGG-triplet sequence, which is the protospacer adjacent motif (PAM) that can be recognized by the *Staphylococcus pyogenes* Cas9 (spCas9) RNP that contains a nuclear localization signal (NLS) to guide the RNP/Cas9 complex to the nucleus to cleave the target sequence. The selected gRNA sequences were further cross-referenced with an HIV sequence database, confirming high levels of conservation (>90%) across HIV-1 sequences from published viral strains (Supplementary Figure S1). The features of these selected gRNAs, including their name, lengths, start/end positions, plus/minus strands, nucleotide sequences, % of ATGC nucleotides, seed GC contents, S-/E-/Doench-scores, and number of mismatch hits, etc., are summarized in Supplementary Table S1. 

### 2.3. Synthetic gRNA/Cas9 RNP Inhibits Viral Replication in Latently HIV-Infected Cell Lines

**Figure 2:**
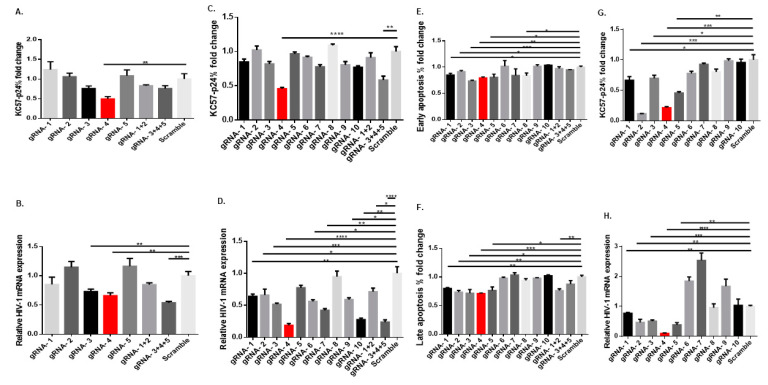
**Treatment with gRNA/Cas9 RNPs reduces HIV-1 reactivation and apoptosis in J1.1 and U1 cells.** (**A**) HIV p24 expression in J1.1 cells transfected with various gRNA/Cas9 RNPs for 48 h, normalized to the scramble control. ** *p* < 0.01. (**B**) Relative HIV mRNA levels in J1.1 cells transfected with various gRNA/Cas9 RNPs for 48 h, normalized to GAPDH, and then, the scramble control. ** *p* < 0.01, *** *p* < 0.001. (**C**) HIV p24 expression in J1.1 cells activated with anti-CD3/CD28 antibodies and transfected with various gRNA/Cas9 RNPs for 48 h, normalized to the scramble control. ** *p* < 0.01, **** *p* < 0.0001. (**D**) Relative HIV mRNA expression in J1.1 cells activated with anti-CD3/CD28 antibodies and transfected with various gRNA/Cas9 RNPs for 48 h, normalized to GAPDH, and then, the scramble control. * *p* < 0.05, ** *p* < 0.01, *** *p* < 0.001, *** *p* < 0.001. (**E**,**F**). Early (Av^+^ 7AAD^−^) and late (Av^+^ 7AAD^+^) apoptosis in J1.1 cells activated with anti-CD3/CD28 antibodies and transfected with various gRNA/Cas9 RNPs 48 h, normalized to the scramble control. * *p* < 0.05, ** *p* < 0.01, *** *p* < 0.001. (**G**) HIV p24 expression in U1 monocytes transfected with various gRNA/Cas9 RNPs for 48 h, normalized to the scramble control. * *p* < 0.05, ** *p* < 0.01, *** *p* < 0.001. (**H**) Relative HIV mRNA levels in U1 monocytes transfected with various gRNA/Cas9 RNPs for 48 h, normalized to GAPDH, and then, the scramble control. ** *p* < 0.01, *** *p* < 0.001, **** *p* < 0.0001.

To test the antiviral effects of our synthetic gRNA/Cas9 RNPs, J1.1 cells were transfected using a published protocol [19]. We tested our gRNAs individually or in combination utilizing a consistent total RNP concentration. Figure 2A shows the fold change of HIV p24 (KC57-p24) antigen expression in J1.1 cells after normalizing KC57-p24% to scramble control, determined by flow cytometry 48 h after gRNA RNP transfection, followed by a 2 h PMA stimulation and 24 h latency reversion. Compared with the scrambled gRNA control, individual and combined gRNAs exhibited varying capability in inhibiting HIV p24 expression, with gRNA-4 (targets HIV-1 *vpr*/*tat* genes) exhibiting the most significant and consistent inhibitory activity. Figure 2B shows the expression of HIV messenger RNA (mRNA; normalized to GAPDH housekeeping gene) in J1.1 cells transfected with gRNA RNPs and stimulated with PMA for viral latency reversal. We used RT-PCR to amplify ~200 bp of mature HIV mRNA with a reverse primer that hybridizes only with poly(A) tail of the spliced HIV mRNA but not with aberrant RNAs and/or integrated/unintegrated proviral DNA [35]. Compared with the scrambled gRNA control, transfection with gRNA-3, gRNA-4, or a combination of gRNA-3 + gRNA-4 + gRNA-5 resulted in a significant reduction in HIV mRNA levels after PMA stimulation. Notably, when used individually or in combination, gRNA-1 and gRNA-2 were highly effective in editing the proviral DNA in vivo when delivered into mice by an AAV system using saCas9-PAM:NGGRR (N) [9] but showed little effects in our transient spCas9-PAM:NGG RNP nucleofection system. Overall, these data illustrate the effectiveness of gRNA/Cas9 gene-editing and the differences between the PAM sequence and the delivery approach.

T cell receptor (TCR) activation can promote transfection efficiency (Supplementary Figure S2A). We thus activated J1.1 cells with anti-CD3/CD28 antibodies in the presence of rhIL-2 for 24 h before gRNA RNP transfection, followed by stimulation with PMA as described above. Similarly, treatment with gRNA-4 or a combination of gRNA-3 + gRNA-4 + gRNA-5 showed a very significant reduction in HIV p24 expression compared with the scrambled gRNA control (Figure 2C). Almost all gRNAs or their combination treatment induced significant decreases in HIV mRNA levels following TCR activation, with gRNA-4 showing the most significant suppression (Figure 2D). Consistent with HIV inhibition, we observed a significant reduction in early (Av^+^7AAD^−^) and late (Av^+^7AAD^+^) apoptosis in J1.1 cells following TCR stimulation, especially in gRNA-4-treated cells compared with the controls (Figure 2E,F). It should be noted that we employed a multi-step nucleofection protocol (i.e., anti-CD3/CD28 stimulation, gRNA/Cas9 nucleofection, and PMA stimulation), which may also trigger cell apoptosis, and thus, the cell apoptosis may not necessarily correlate only with the viral inhibition by our treatment.

Latently HIV-infected U1 monocytes were also employed to confirm the antiviral efficiency of the gRNA/Cas9 RNPs. Figure 2G,H shows p24 and HIV-1 mRNA expression in U1 monocytes transfected with different gRNA RNPs for 48 h and stimulated with TNFα (25 ng/mL) plus PMA (20 ng/mL) for 24 h. All gRNAs decreased the levels of p24 and HIV-1 mRNA, with gRNA-4 inducing the most significant inhibition. Together, these results demonstrate that a one-time gRNA/Cas9 RNP treatment can efficiently reduce HIV reactivation/replication in latently infected J1.1 T cells and U1 monocytes and that gRNA-4 (targeting *vpr*/*tat* genes) shows the most potent and consistent antiviral effect among the single RNPs used. 

### 2.4. Synthetic gRNA-3 and gRNA-4 RNPs Induce a Sustained Antiviral Effect in Latent HIV J1.1 T Cells

**Figure 3:**
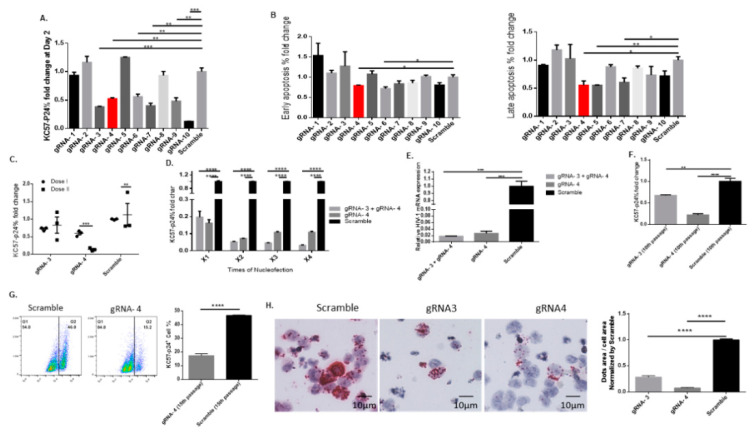
**Treatment with gRNA-3 or gRNA-4 RNP inhibits HIV infection.** (**A**) HIV p24 expression levels in highly permissible SupT1 cells 48 h after incubation with supernatants of J1.1 cells transfected with various gRNA/Cas9 RNPs for 48 h, normalized to the scramble control. ** *p* < 0.01, *** *p* < 0.001. (**B**) Early and late apoptotic SupT1 cells infected by the supernatants of J1.1 cells transfected by various gRNA/Cas9 RNPs for 48 h, normalized to the scramble control. * *p* < 0.05, ** *p* < 0.01. (**C**) HIV p24 expression in J1.1 cells transfected with one dose of gRNA-3 or gRNA-4 RNP (dose I, round symbol). Then, the cells were sorted and transfected with a second dose gRNA-3 or gRNA- 4 RNP for 48 h (dose II, square symbol), normalized to the scramble control. *** *p* < 0.001. (**D**) HIV-1 p24 expression after a dose-dependent (X1, X2, X3, X4) RNP treatment/transfection of J1.1 cells for 1, 5, 10, and 20 days with combined (gRNA-3 + gRNA-4) or single gRNAs (gRNA-4 or Scramble). **** *p* < 0.0001. (**E**)**.** HIV-1 mRNA levels in total RNA isolated after four doses of nucleofection. (**F**) HIV p24 expression in J1.1 cells transfected with gRNA-3 or gRNA-4 RNP and maintained for 10 passages, normalized to the scramble control. ** *p* < 0.01, **** *p* < 0.0001. (**G**) HIV p24 expression in J1.1 cells transfected with the gRNA-4 RNP and maintained for 15 passages, normalized to the scramble control. **** *p* < 0.0001. (**H**) HIV-1 RNA levels in J1.1 cells transfected with gRNA-3 or gRNA-4 RNP, assessed by RNAscope. Representative RNAscope imaging (left) and summary data (right) were analyzed by QuPath software (bundled with 64-bit Java 1.8.0_172) and GraphPad Prism (Version 6.01) to quantify the positive HIV RNA dots in 6 imaging areas, normalized to the scramble control. **** *p* < 0.0001.

To determine whether the gRNA/Cas9-treated J1.1 cells produce less infectious viral particles, approximately 100 µL of the cell supernatants (from the experiments depicted in Figure 2C,D) were used to infect highly permissible SupT1 cells according to our previously published spinoculation methods [27]. As shown in Figure 3A, the SupT1 cells infected with supernatants from several gRNA/Cas9 RNP-treated cells (including gRNA-3, gRNA-4, gRNA-6, gRNA-7, and gRNA-10) showed reduced viral infection at 48 h after spinoculation, as evidenced by the significant reduction in HIV p24 protein expression percentage normalized to and compared to cells infected with supernatant from scramble gRNA-treated cells. Correspondingly, incubation of SupT1 cells for 48 h with supernatants of J1.1 cells treated with gRNA/Cas9 RNPs (including gRNA-4) resulted in a significant decrease in early and late apoptosis (Figure 3B). These data suggest that gRNA-4 treatment of latently HIV-infected J1.1 cells reduces the production of infectious HIV particles. 

We next sought to determine whether gRNA/Cas9 RNP sustains its antiviral activity when repeatedly treating HIV-infected cells with the same gRNA/Cas9 RNP complex. To this end, J1.1 cells were activated with anti-CD3/CD28 antibodies in the presence of rhIL-2 for 24 h, transfected with gRNA/Cas9 RNPs for 48 h, stimulated with PMA for 2 h, and cultured for an additional 24 h (Dose I). The cells were then subjected to the same treatment for a second round (Dose II). Only gRNA-3 and gRNA-4 RNPs were used in this experiment. Figure 3C shows that Dose II treatment with gRNA-4 RNP significantly (90% inhibition compared to scramble control) reduced HIV p24 expression compared to Dose I, whereas the second treatment using gRNA-3 RNP had no such an effect, possibly due to a mismatch binding site after Dose I treatment with viral DNA cleavage and damage repair and thus escape. To improve the antiviral effect, we performed a dose-dependent combination treatment with gRNA-3 + gRNA-4 and a single treatment with gRNA-4 or scramble control. Figure 3D shows a reduction in p24 protein levels following nucleofection with multiple doses. Complete virus inactivation is required for an HIV cure. However, even after three or four nucleofections and combination gRNA treatment, we could not achieve a 100% elimination of viral replication. Ultimately, we observed a 95–98% reduction in HIV p24 expression (Figure 3D) as well as HIV mRNA levels (Figure 3E) with these treatments compared to the scramble control. These data suggest that achieving a 100% reduction in viral replication might not be feasible due to various limitations, such as nucleofection efficiency, gene-editing, individual gRNAs/CRISPR efficiency [36], and new virus production and re-infection of new (or edited) cells during the treatment.

Additionally, we sought to establish a stable cell line by transfecting J1.1 cells using GFP-labelled gRNA RNP and then sorting the GFP^+^ cells (Supplementary Figure S2B). The sorted GFP^+^ cells were cultured for a long period of time (10 passages) and HIV p24 expression was assessed following viral induction by PMA stimulation. As shown in Figure 3F, gRNA-3 and gRNA-4-treated cells exhibited a significant decrease in p24^+^ cells (25% and 75% inhibition, respectively) compared with the scramble gRNA-treated cells. We also assessed p24 expression in J1.1 cells after 15 passages and found a sustained viral (p24) inhibition by the gRNA-4 RNP treatment (Figure 3G). Moreover, we measured HIV RNA alterations in J1.1 cells stably transfected with gRNA RNPs using RNAscope, which utilizes the RNA in situ hybridization technique. As shown in Figure 3H, HIV RNA was significantly inhibited by gRNA-3 and especially gRNA-4 RNP treatment. These data indicate that gRNA/Cas9 RNPs can exert a sustained antiviral effect, with a maximum of ~95–98% viral inhibition in latently HIV-infected cells, in a dose- and time-dependent manner. However, this approach did not completely (i.e., 100%) abolish the HIV reactivation/replication, even with multiple doses and gRNA combination treatments. 

### 2.5. On-Target Gene-Editing Capability and Off-Target Cytotoxic Effects of Synthetic gRNA/Cas9 RNPs

**Figure 4:**
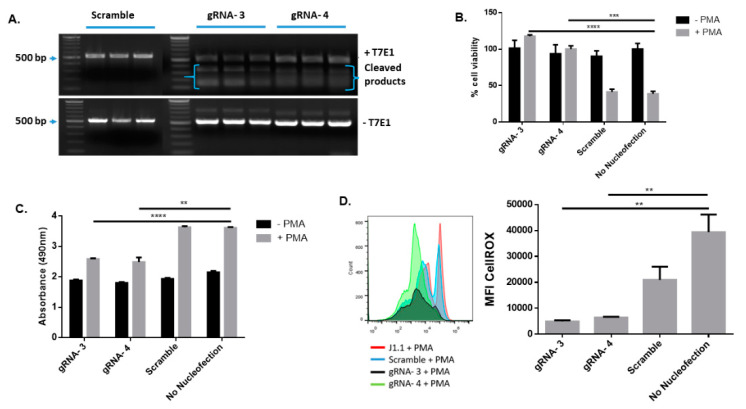
**Mismatch mutagenesis, cellular cytotoxicity, and ROS production in J1.1 cells treated with gRNA-3 or gRNA-4 RNP.** (**A**) The mismatch mutagenesis was assessed by the T7E1 assay. The T7E1-cleaved HIV DNA fragments in gRNA-3 or gRNA-4 RNP-treated cells were analyzed by standard PCR. (**B**) Cellular viability of gRNA RNP-treated and untreated J1.1 cells was measured by the MTT assay. *** *p* < 0.001, **** *p* < 0.0001. (**C**) LDH release from the treated and untreated cells was measured using the LDH release assay. ** *p* < 0.01, **** *p* < 0.0001. (**D**) CellROX assay was performed to assess ROS level in gRNA-3 or gRNA-4 RNP-treated cells in the absence or presence of PMA stimulation for 24 h. Representative histogram and the summary data of CellROX MFI are shown. ** *p* < 0.01.

DNA cleavage by CRISPR/Cas9 leads to double-strand breaks (DSBs). DSBs in mammalian cells are primarily repaired by either the non-homologous end joining (NHEJ) and/or homologous recombination (HR) repair pathways [9,10,11,37]. The NHEJ repair is initiated following genomic editing by CRISPR and introduces insertion/deletion (indel) mutations [38,39], whereas the HR repair is induced in a high-fidelity, template-dependent DNA repair following complex DNA damages such as DNA gaps, DSBs, and DNA interstrand crosslinks [40,41]. Thus, evaluation of the DNA cleavage and insertion/deletion (indel) mutations following CRISPR/Cas9-mediated DNA damage and repair is important to confirm its gene-editing capability. We performed this evaluation using a T7 endonuclease 1 (T7E1) mismatch cleavage assay after PCR amplification of the DNA regions that flank each gRNA-targeting site using specific primers (Table 1). The size of these DNA fragments depends on the distance between each primer’s location on target genes as well as the gRNA cleavage site. The gene-editing, mismatch repair, and the Indel mutation events were detected after the gRNA/Cas9 RNP-mediated proviral DNA cleavage, HIV DNA PCR-amplification, and digestion by the T7E1 nuclease. As shown in Figure 4A, the cleaved mismatched fragments of HIV DNA shown in the upper panel (+T7E1) for both gRNA3- and gRNA4-treated J1.1 cells indicated the presence of gene-editing and mismatch repair in HIV DNA. However, these cleaved mismatched DNA fragments were not detected in the T7E1-untreated (-T7E1) or scramble gRNA-treated cells. These data indicate that gRNA-3 RNP and gRNA-4 RNPs can induce specific HIV DNA cleavages, which can be detected by the T7E1 mismatch cleavage assay. 

We further confirmed the gRNA/Cas9 RNP-mediated HIV-1 DNA cleavage by performing a Sanger sequencing after PCR amplification of the DNA fragment containing gRNA binding sites using appropriate primers (Table 2). Following a successful CRISPR/Cas9 cleavage, DNA damage repair pathways can initiate and substitute/add random bases, thus introducing indel mutations. These changes affect the DNA sequencing readout by capillary electrophoresis, because the DNA analysis cannot determine the nucleic acid sequence after it detects/reaches the gRNA cleavage site. As such, an internal cut could technically stop a sequence reading signal abruptly. We observed this following nucleofection and extraction of genomic DNA from the treated J.1.1 cells, where the sequencing readout displayed a high level of “noise” beyond the cleavage site for HIV-gRNA-treated cells, but not for the scramble gRNA control group (Supplementary Figures S3 and S4).

NAD(P)H-dependent cellular oxidoreductase enzymes reduce the tetrazolium MTT (3-(4,5-Dimethylthiazol 2-yl)-2,5-diphenyltetrazolium bromide) salt into insoluble formazan, which indicates the overall survival and metabolic activity of a living cell. To determine whether gRNA/Cas9 RNPs exert cytotoxic effects, we measured the percentage of cell viability by MTT assay at 24 h after transfection of J1.1 cells with gRNA-3 and gRNA-4 RNPs. In the absence of PMA stimulation, we did not observe any significant changes in cell viability among treatment groups compared to the untreated cells, suggesting the nucleofection protocol does not induce cytotoxicity. Notably, following PMA stimulation, the cell viability was decreased in scramble gRNA-treated cells and no nucleofection control (likely due to the pathologic apoptotic effect of HIV replication), but not in gRNA-3- or gRNA-4-treated cells, indicating that the treatment inhibited HIV reactivation/replication-mediated cell death without inducing significant cellular cytotoxicity (Figure 4B). 

Lactate dehydrogenase (LDH) is a cellular enzyme that is quickly released into the culture medium upon cell damage and is widely used for quantitative measurement of cellular cytotoxicity. Using this method, we measured LDH release from J1.1 cells transfected with gRNA-3 or gRNA-4. We did not observe any significant differences in cellular LDH release in the absence of PMA stimulation (i.e., HIV replication), indicating a lack of cytotoxic effects due to the nucleofection protocol. LDH release significantly increased following PMA stimulation (or HIV replication), but its levels were lower in gRNA-3- and gRNA-4-treated cells compared with the scramble control treatment and no nucleofection control, indicating the ability of gRNA-3- and gRNA-4 RNPs to inhibit HIV reactivation/replication-induced cell death without causing significant cytotoxic effects (Figure 4C). 

We have previously shown that HIV infection depletes CD4 T cells by increasing telomeric DNA damage and apoptosis via increasing cellular reactive oxygen species (ROS) production and inducing mitochondrial dysfunction [27,42,43,44]. To determine whether gRNA-3 or gRNA-4 RNPs can inhibit HIV reactivation/replication while maintaining cell survival via inhibiting ROS production in PMA-activated cells, we measured the mean fluorescence intensity (MFI) of CellROX (a readout for cellular ROS production). As shown in Figure 4D, the MFI of CellROX was significantly decreased in gRNA-3- and gRNA-4-treated J.1.1 cells compared with the scramble and no-nucleofection controls. However, a reduction in the MFI was observed with the scramble control when compared to the no-nucleofection control. This effect is solely caused by nucleofection, as anti-CD3/CD28 stimulation and nucleofection can increase cell apoptosis. This correlation between apoptosis and CellROX MFI was addressed in our previous work [45]. Taken together, these results indicate that the gRNA-3 and gRNA-4 RNP treatment can cleave HIV DNA and alter cell viability and ROS production by inhibiting HIV reactivation/replication, aiding cellular survival and well-being.

### 2.6. Effects of Synthetic gRNA/Cas9 on Cell Activation, Apoptosis, and DNA Damage Repair Pathways

**Figure 5:**
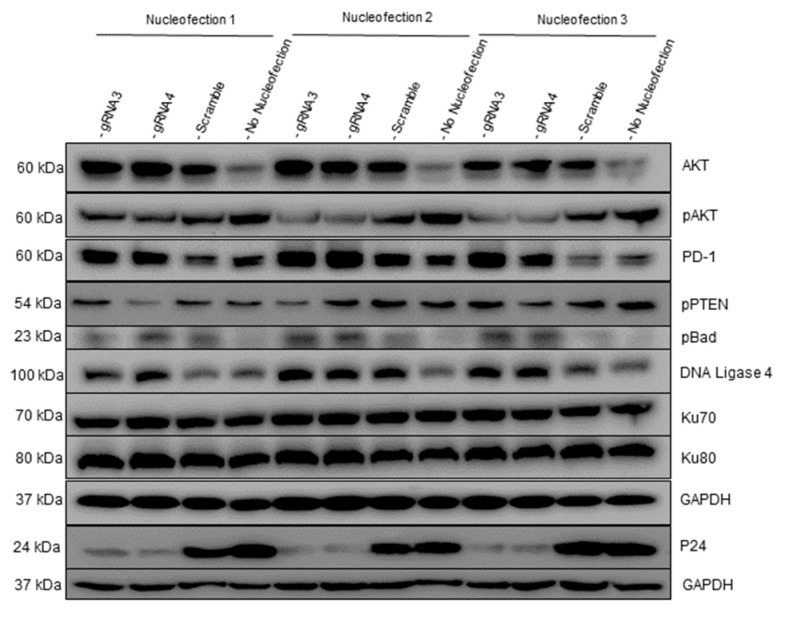
**Cell activation, apoptosis, and DNA damage repair molecules in J1.1 cells treated with gRNA-3 RNP or gRNA-4 RNP.** Representative Western blots showing AKT, pAKT^Ser473^, PD-1, pPTEN^Ser370^, pBad^Ser112^, DNA ligase 4, Ku70, Ku80, P24, and GAPDH proteins in J1.1 cells after PMA stimulation, followed by the transfection using gRNA-3, gRNA-4, or scramble gRNA RNP. Nucleofection 1, 2, and 3 represent three independent experiments. For no nucleofection samples (last lane), non-transfected J1.1 cells were stimulated with PMA.

To further evaluate the effects of gRNA-3 or gRNA-4 RNPs on HIV-infected cells, we performed Western blot to analyze the expression of molecules involved in cell activation, apoptosis, and DNA damage repair pathways in PMA-stimulated J1.1 cells following gRNA-3 and gRNA-4 RNP treatment. We have previously shown that HIV infection alters the expression and activation (phosphorylation) of AKT—an important signaling molecule in the TCR signaling pathway [27,42]. Interestingly, we found that the level of total AKT protein was upregulated in gRNA-3- or gRNA-4-treated cells compared with the scramble gRNA control or no nucleofection treatment (Figure 5). However, pAKT^Ser473^ expression was downregulated in the gRNA-3- or gRNA-4-treated cells compared with the scramble gRNA control or no nucleofection treatment. These results indicate that cell activation and HIV replication inhibit total AKT protein expression and increase pAKT levels, whereas inhibition of HIV replication by gRNA-3 and gRNA-4 RNPs alleviates the HIV-mediated suppression of total AKT expression and prevents cell overactivation and subsequent cell apoptosis.

PD-1 is an inhibitory molecule (and also an activation marker) in the TCR signaling pathway. We have previously shown that PD-1 expression is significantly altered on CD4 T cells during HIV infection [42,43]. We found that gRNA-3 and gRNA-4 RNP treatment upregulated the PD-1 level in J1.1 cells. In addition, the phosphorylation of PTEN^Ser380^ (a lipid phosphatase that acts as a tumor suppressor by targeting the PI3K/AKT pathway) was inhibited in gRNA-4-treated cells compared to the controls, indicating HIV-induced activation of the PI3K/AKT signaling is attenuated in gRNA-4-treated cells (Figure 5). 

We have previously shown that HIV drives CD4 T cell depletion via regulating the expression of apoptotic molecules, including anti-apoptotic pBAD [42]. We found that pBAD was upregulated in gRNA-3 and gRNA-4 RNP-treated cells compared to the control groups, which is consistent with our previous report showing that active HIV infection induces T cell apoptosis through downregulation of pBAD [42].

Because gRNA-3 and gRNA-4 RNP treatments did not achieve complete inhibition of HIV replication, likely due to either the low nucleofection efficiency or even (if less likely) viral mutation/escape, we examined the expression of molecules involved in DNA damage repair in J1.1 cells, following the cleavage of HIV DNA by gRNA-3 and gRNA-4 RNPs. Notably, DNA ligase-4 was upregulated in the gRNA-3- and gRNA-4-treated cells compared with the controls; however, the Ku70 and Ku80-related DNA repair pathways were not altered (Figure 5). The Ku70/Ku80 heterodimer is a major component and a central player in the NHEJ repair pathway [46]. Similarly, the DNA ligase-4 is also important component in the NHEJ pathway and required to ligate and repair the DSBs [47]. The increase in DNA ligase-4 expression in the gRNA-3- and gRNA-4-treated cells indicates a process of gene-editing and ligation, which triggers activation of the NHEJ DNA repair pathway. Surprisingly, Ku70 and Ku80 expression was not altered by the CRISPR treatment, which warrants further kinetic studies. As expected, we observed a significant decrease in p24 protein levels in gRNA-3 and gRNA-4 treated cells compared to the control groups. 

## 3. Discussion

In the present study, we used synthetic gRNA RNP complexes as non-viral formulations as a presage to developing CRISPR-Cas9-mediated HIV therapeutic drugs. We designed a series of gRNAs targeting different HIV genes crucial for viral replication and tested their antiviral efficacy and cellular cytotoxicity in latent HIV cell lines. We demonstrated that these synthetic HIV gRNA/Cas9 RNPs can efficiently suppress HIV reactivation and replication, as evidenced by a significant inhibition of HIV RNA and antigen expression levels. While the detailed molecular mechanisms underlying HIV suppression by gRNA/Cas9 RNPs are being characterized, our data support that these drugs can canonically induce a rapid cleavage of the integrated HIV DNA, an immediate response from DNA damage and repair, resulting in a high percentage of indel mutations that collapse viral gene reactivation and replication. These findings demonstrate that our synthetic gRNA/Cas9 RNPs have the potential to be employed as novel HIV-targeting drugs capable of promoting HIV eradication. 

The establishment and maintenance of HIV-1 latent reservoirs are associated with various host-cell markers, such as PD-1, Bim, TIGIT, LAG-3, CTLA-4, CD2, CD-20, CD30, and CD32a, making them potential candidates for targeting HIV reservoirs [48,49,50,51,52,53,54,55]. However, such approaches have proven ineffective in eliminating latent HIV reservoirs, and to date, how HIV-1 is incorporated into the human genome to establish viral latency and its relationship with the initiation of ART remain unclear [56,57,58,59,60]. In addition, there have been several other attempts to achieve a “sterilized and functional HIV cure”. For example, a short interfering RNA (siRNA) approach has been used to clear HIV reservoirs by targeting the anti-apoptotic SAF lncRNAs in macrophages [61]. The use of engineered anti-HIV chimeric antigen receptor T (CAR-T) cells to kill reservoir cells is another new technique to cure HIV latency [62,63,64]. Other ongoing and promising approaches include strategies such as “kick and kill”, “block and lock”, gene-editing using CRISPR, Zinc finger nucleases (ZFNs), combinatory therapeutic T-cell vaccines triggering CTL and other immune cell responses, use of passive neutralizing antibodies, and immune checkpoint inhibitors [65]. Despite these efforts, eliminating latent HIV reservoirs is still an existing unmet medical need. Thus far, HIV has only been cured in two individuals with hematological malignancy treated with hematopoietic stem cell transplantation using donors with homozygous CCR5Δ32 mutation—identified as the “Berlin patient” and “London patient”—where the mutant CCR5Δ32 prevented further HIV progression [66,67,68]. While still early into evaluation, a third patient (the “New York patient”) is another cure based on a similar approach. However, this approach is not suitable or feasible for broad clinical applications due to the high morbidity and mortality associated with stem cell transplantation and the potential of HIV co-receptor switching from CCR5 to CXCR4 or CCR2 [69,70]. 

Synthetic gRNA/Cas9 RNPs can induce rapid DNA cleavage, with reduced off-target effects, low risk of insertional mutagenesis, easy production, and readiness for use in clinical applications [18,71]. Administration of such synthetic gRNA/Cas9 RNPs can offer a direct and transient gene-editing effect in a controllable way compared with other delivery methods that use plasmid DNA (pDNA) or mRNA, which exhibit varying transcriptional/translational activity and uncontrolled expression duration in vivo and thus can result in off-target effects and unwanted immune responses [72]. In this study, we selected 10 gRNA target sites on the HIV genome with the highest levels of conservation among viral strains and the lowest overlap (off-target) with the host genome. We found that gRNA-4, targeting the critical HIV-1 *vpr*/*tat* genes, exhibited the most potent activity in inhibiting HIV reactivation and replication. We propose that these HIV genomic regions are optimal target sites for the CRISPR/Cas9 RNP approach, which warrants further investigation for drug development to eradicate HIV infection. 

Despite using the E-CRISP gRNA design to select specific target genes critical for HIV replication, the antiviral effect of individual gRNAs varied between J1.1 and U1 cells. This is most likely due to the differences in cell type and stimulation conditions. The cleavage efficiency of a gRNA is dependent on multiple variables, such as cell type, gRNA/Cas9 complex formation, targeted sequence location, binding affinity, GC content, chromatin accessibility, gene expression, RNA secondary structure, melting temperature, and free energy [36]. In addition, the latent HIV genome in these cell lines may introduce potential mutations over time, which could lead to sequence mismatch and thus non-functional gRNAs. Nonetheless, gRNA-4 showed consistent and significant antiviral effects in both J1.1 and U1 cell lines.

Evaluation of the DNA cleavage and indel mutations following CRISPR/Cas9-mediated DNA damage and repair is important to confirm its on target gene-editing. We performed this evaluation using a T7E1 mismatch cleavage assay after PCR amplification of the DNA fragments that flank each gRNA-targeting site. The next step of the verification process is to use Sanger sequencing to verify that the intended region has been edited/deleted. However, this step can be slightly tricky by the fact that the CRISPR-Cas9 system rarely modifies the DNA at exactly the nucleotide expected. Often, it will cut the DNA up to three base pairs away from the excision site. This is problematic for Sanger sequencing because it can create a mixture of products that vary by one, two, or three nucleotides in length. In practice, this means that the chromatogram before the cleavage site can be read easily, but after the editing/deletion site, the sequencing trace will give scrambled results, as we observed and shown in Supplementary Figures S3 and S4. The workaround is to sequence the PCR product in both the forward and reverse directions, in order to map both upstream and downstream of the gene editing and deletion.

Although we did not observe any significant cytotoxic effects by our gRNA/Cas9 RNPs, off-target effects are always a concern due to target mismatch by CRISPR/Cas9. Any unexpected off-target effects could cause structural chromosomal rearrangements in the host cell, which may result in oncogene activation or genome instability [73,74,75,76]. Thus, this study warrants further investigation into any potential off-target effects in CRISPR-mediated gene editing and cleavage detection and could benefit from the use of cutting-edge techniques, such as anchored primer enrichment (GUIDE-seq), in situ detection (BLISS), in vitro selection libraries (CIRCLE-seq), chromatin immunoprecipitation (ChIP), DISCOVER-Seq, translocation sequencing (LAM PCR HTGTS), and in vitro genomic DNA digestion (Digenome-seq and SITE-Seq), as described by Atkins et al. [77]. In addition, the risk of germline transmission is also a critical challenge to the safety of this approach. Thus, the risks associated with this new technology must be fully investigated and resolved before its clinical application. 

Interestingly, we observed that PD-1 protein expression was upregulated in gRNA/Cas9 RNP-treated J1.1 cells compared with the untreated or scramble gRNA/Cas9 RNP-treated cells, whereas pAKT and pPTEN levels were downregulated. These results indicate that HIV infection inhibits total AKT and PD-1 protein expression but activates the PI3K/AKT signaling pathway. Thus, inhibiting HIV replication could alleviate virus-mediated AKT and PD-1 inhibition and prevent T cell overactivation and thus apoptosis. PD-1 upregulation and pPTEN downregulation in gRNA/Cas9 RNP-treated cells also indicate the initiation of DNA DSB repair pathways and the presence of genomic instability. Additionally, we found that pBAD^Ser112^ was upregulated in the gRNA/Cas9RNP-treated J1.1 cells. These results support our previous findings in HIV infection [42] that while BAD protein is pro-apoptotic, phosphorylated BAD (pBAD) is anti-apoptotic. This likely occurs because canonical anti-apoptotic proteins such as BCL-2/BCL-XL form heterodimers with BAD during BAD-dephosphorylation, and this phenomenon can trigger BAX/BAK-mediated apoptosis. However, when BAD is phosphorylated by AKT and BCL-2 is released, this free BCL-2 can inhibit BAX-triggered apoptosis [78]. It is highly likely that HIV inhibition by our gRNA/Cas9 RNPs alleviated the suppressive effects on cellular survival factors, which inhibit the pro-apoptotic activity of Bad protein by activating intracellular signaling pathways that result in the phosphorylation of Bad at Ser112 and Ser136 [79]. These events thus led to increases in the expression levels of pBAD in gRNA-3- and gRNA-4/Cas9 RNP-treated cells compared with the control groups. Notably, these findings are in line with our observation that apoptosis was reduced in gRNA-3 and gRNA-4 RNP-treated cells and that the levels of HIV viral particles were lower. Surprisingly, while DNA ligase-4 level was increased in gRNA-3 and gRNA-4 RNP-treated cells, we did not observe any marked changes in Ku70 and Ku80 protein levels, suggesting limited activation of the DNA repair pathways. It should be pointed out that we used cells that have been transfected for a long period of time (10 passages after gene editing) for Western blot analysis. DNA repairs by Ku proteins are, however, likely to occur in cells experiencing DNA damage at early time points following CRISPR/Cas9-mediated gene-editing [37,80]. 

This study has some limitations, such as the lack of evaluation of the long-term inhibitory effects of gRNA/Cas9 RNPs and comprehensive analysis of the antiviral activity as well as the host in vivo immune responses. The nucleofection efficiency is a major obstacle because it cannot deliver the RNPs into every latently infected cell. A previous study on nucleofection efficiency using different cell types justifies our limitation of achieving a 100% nucleofection [81]. This will permit untreated cells to produce new viral progeny, which can infect/reinfect other cells including those cells that are already edited. Previous studies indicated that virus escape due to using only one gRNA can reduce HIV-1 elimination [12,38,39,82,83] and that a combination of gRNAs can improve viral elimination [12,84,85,86,87]. Previous studies reported efficient inhibition or elimination of HIV replication using gRNA-expressing vectors and/or Cas-expressing vectors with appropriate delivery methods (i.e., by lentivirus and AAV) [9,88,89,90,91,92,93]. Using a synthetic, transient RNP formulation is a fairly new approach in HIV eradication research [12]. Unlike stable or long-term expression systems, which may potentially lead to oncogenesis, off-targeting, and unwanted immune responses, a transient RNP will not have any effects on new generations of replicating cells and will be degraded over time. These factors should be taken into consideration when formulating future antivirals for treating HIV patients. In this study, even after attempting multiple dose- and time-dependent nucleofection protocols, dual gRNA (gRNA-3 + gRNA-4) combination treatment, and Cas9-GFP cell sorting to establish a stably gene-edited cell line, we were unable to achieve a complete (100%) elimination of the p24 antigen expression or eradication of mature HIV-1 mRNA, and we believe this is mainly due to nucleofection efficiency. We would expect that developing a novel nanoparticle or exosom-based drug delivery vehicle would improve the in vivo efficacy when using this synthetic gRNA/Cas9 therapeutic system in conjunction with ART to cure HIV.

In summary, this study provides a proof-of-concept that synthetic gRNA/Cas9 RNPs can efficiently inhibit HIV reactivation/replication. Further development of multiplexed gRNA/Cas9 RNPs using engineered nanoparticles [20] or exosomes [21] for drug delivery may overcome these challenges and facilitate their clinical application. Humanized HIV-infected murine models offer a good opportunity for further evaluating gRNA/Cas9 RNP antiviral activity and side effects in vivo.

## 4. Materials and Methods

**Cell culture and latency reversal**. Jurkat E6-1 (J1.1) T cells (latently infected with HIV-1 and cloned by limiting dilution) [24] and U1 monocytes (cloned by chronic HIV-1 infection of promonocytes) [29] were obtained from the NIH AIDS Reagent Program (J1.1 cell line was originally from Dr. Thomas Folk, and the uninfected parental Jurkat E6-1 clone was from Dr. Arthur Weiss) [24,29,94]. The cells were cultured in complete RPMI 1640 medium supplemented with 10% FBS (Atlanta Biologicals, Flowery Branch, GA, USA), 100 IU/mL penicillin, and 2 mM L-glutamine (Thermo Scientific, Logan, UT, USA) and incubated at 37 °C in a 5% CO_2_ atmosphere. For HIV latency reversal, J1.1 cells were stimulated one-time with 20 ng/mL PMA (Thermo Fisher Scientific, Waltham, MA, USA) for 2 h, and U1 monocytes were stimulated with both PMA (20 ng/mL) and TNFα (25 ng/mL) for 24 h. Cells were harvested at 24 h, 48 h, and 72 h after the stimulation for further analysis.

**Flow cytometry.** Cellular apoptosis was assessed using the Apoptosis Detection Kit I (BD Biosciences, San Jose, CA, USA). Briefly, the cells were harvested at different times after transfection with gRNA RNPs, washed with DPBS, stained with Av and 7AAD, and analyzed by flow cytometry. HIV reversal was assessed by intracellular staining with a PE-conjugated anti-HIV-1 p24 monoclonal antibody (Clone KC57, Beckman Coulter, Brea, CA, USA) to measure HIV rebound. The intracellular staining was performed after permeabilization with fixation/permeabilization reagents (Thermo Fisher Scientific), and the cells were analyzed by AccuriC6 Plus flow cytometer (BD Biosciences) with FlowJo software (Tree Star, Ashland, OR, USA). Single staining, isotype control antibodies, and unstained control were used to determine the staining background levels and adjust multicolor compensation as a gating strategy.

CellROX green reagent (Cat# C10444, Thermo Fisher) was used to measure cellular oxidative stress. The gRNA RNP-treated cells were incubated with CellROX dye (final concentration 2.5 mM) at 37 °C for 30 min. The cells were washed three times with PBS and analyzed by flow cytometry to determine ROS levels. When oxidized by reactive oxygen species (ROS), CellROX dye binds to DNA and exhibits a bright green fluorescence color at ~500 nm absorbance.

**gRNA design and synthesis.** The gRNAs were designed using the E-CRISP online tool based on the HIV-1 sequence (strain LAV-1, bases 1-9709) derived from the HIV-1 vector PNL4-3 (HIVPNL4-3) sequence (PubMed Accession no. AF324493.2) [95]. As previously described, both J1.1 and U1 cell lines were cloned using the HIV-1 (LAV-1) strain [24,29,30]. E-CRISP provides and compares distinct gRNAs based on the parameters of target specificity, efficacy, mismatch hits, and characteristics of the selected gRNAs per Doench score [32,33] and Xu [34] score, which are calculated by the E-CRISP online tool. The sequence conservation was obtained using the NCBI nucleotide blast tool, and alignment was performed using the Clustal Omega multiple sequence alignment tool from EMBL-EBI. The selected gRNAs were synthesized by Sigma-Aldrich (St. Louis, MO, USA) and purified by HPLC.

**CRISPR/Cas9 RNP complex formation and transfection.***Staphylococcus pyogenes* Cas9 Nuclease protein with nuclear localization signal (NLS) and the PAM recognition site (NGG) was purchased from Horizon Discovery and Macrolab from the University of California (Berkley). The GFP-labelled Cas9 protein (Cas9 GFPPRO) was obtained from MilliporeSigma. The scramble gRNA and tracrRNA were obtained from Horizon Discovery. Briefly, 160 µM gRNA and 160 µM tracrRNA were mixed and incubated at 37 °C for 30 min to form a gRNA:tracrRNA duplex. Approximately 80 µM of the RNA duplex was mixed with 40 µM Cas9 protein and the mixture was incubated at 37 °C for 15 min. This gRNA/Cas9 complex was used to transfect 5 × 10^6^ target cells by nucleofection, using the method described by Hultquist et al. [19]. The SE cell line 4D-NucleofectorTM X Kit L (Lonza, Basel, Switzerland) was used to perform the nucleofection in a 4D nucleofector core unit using the unique program for E6-1 cells. For cell stimulation, the cells were incubated with 1 µg/mL each of anti-CD3/anti-CD28 (BD Biosciences) and 100 IU IL-2 for 24 h before nucleofection. After nucleofection, the cells were cultured for 48 h in complete RPMI 1640 medium, stimulated with 20 ng/mL PMA for 2 h, washed twice, and then cultured in complete RPMI 1640 medium for an additional 24 h.

**RT-PCR.** To measure HIV-1 mRNA levels, total RNA was extracted from 2 × 10^6^ gRNA RNP-treated cells using PureLink RNA Mini Kit (Invitrogen, Carlsbad, CA, USA). The cDNA was synthesized from 100 ng total RNA using the High Capacity Reverse Transcription Kit (Applied Biosystems, Foster City, CA, USA). Approximately 1 µL cDNA was used to perform PCR using iTaq Universal SYBR green master mix from Bio-Rad. The primers used in the PCR were: HIV mRNA forward 5′-CAGATCCTGCATATAAGCAGCTG-3′ and reverse 5′-TTTTTTTTTTTTTTTTTTTTTTTTGAAGCAC-3′; GAPDH mRNA 5′-TGCACCACCAACTGCTTAGC-3′ and reverse 5′-GGCATGGACTGTGGTCATGAG-3′. The PCR reactions were run in triplicate under the following conditions: 1 cycle at 95 °C for 10 min, followed by 35 cycles at 95 °C for 15 s, 60 °C for 60 s, and 72 °C for 60 s. Gene expression levels were calculated using the 2^−ΔΔct^ threshold method. The values were first normalized to the GAPDH level and then to the control (scramble) group and are presented as fold change.

**Mismatch cleavage assay**. We used the T7 Endonuclease 1 (T7E1) assay to detect on-target HIV gene-editing in gRNA RNP-treated J1.1 cells. This assay helps confirm specific DNA cleavage and repair within the gRNA-targeting region of the genome. Briefly, we isolated genomic DNA from each treatment and control group and then amplified the gRNA-flanking region in the proviral genome by real-time PCR using specific primers (Table 1). The PCR product was denatured at 95 °C for 5 min and slowly reannealed using cooling ramp rates to form a heteroduplex mismatch at regions where DSBs have occurred. The cooling ramp rates were 95–85 °C, at −2°C/second and 85–25 °C, at −0.1°C/second. These mismatches are recognized and cleaved by T7E1 digestion (5 units per reaction, Cat# E3321, New England Biolabs, Ipswich, MA, USA), and the cleaved DNA was detected using 1% agarose gel electrophoresis. Images of the cleaved DNA fragments were acquired using a gel imaging system (Bio-Rad, Hercules, CA, USA).

**Proviral DNA sequence analysis.** Following the 4th dose of RNP treatment, genomic DNA was isolated from J1.1 cells using PureLink Genomic DNA Mini Kit (Cat# K182001, Invitrogen). The DNA fragments containing gRNA targets were amplified by PCR using primers (Table 2) flanking the cleavage site (~200 bp on both forward and reverse end). The PCR products were confirmed using 1% agarose gel electrophoresis and purified using a QIAquick gel extraction kit (Cat# 28704, Qiagen). The extracted DNA fragments were sequenced by Sanger sequencing with appropriate primers (Table 2), at the ETSU molecular biology core facility. The sequencing readout were aligned and compared to the scramble control, which is identical to the HIV-1 sequence. The Chromas tool (Technelysium DNA sequencing software) was used to read the nucleotide peaks, and the Clustal Omega multiple sequence alignment tool from EMBL-EBI was used to align and compare the nucleotide sequences of our PCR products.

**MTT assay.** MTT assay was performed using a commercially available kit (Cat# 465007001, Roche, Basel, Switzerland). Briefly, J1.1 cells were cultured in a 100 μL RPMI 1640 culture medium with or without PMA stimulation for 2 h and then rescued for 24 h in a 96-well plate. MTT labeling reagent was added at a final concentration of 0.5 mg/mL. The cells were incubated at 37 °C for 4 h, followed by adding the solubilization buffer and incubating at 37 °C overnight. The MTT absorbance was measured at 550 nm using a spectrophotometer (MilliporeSigma, Burlington, MA, USA).

**LDH assay.** LDH release was measured using a commercially available kit (Cat# 11644793001, Roche). Briefly, J1.1 cells were cultured in 100 μL RPMI 1640 medium with or without PMA stimulation for 2 h, rescued for 24 h, and then treated with the LDH dye at 37 °C for 30 min. A colorimetric measurement was performed using a spectrophotometer (MilliporeSigma) at 490 nm absorbance. A high level of absorbance suggests an increase in LDH release, thus indicating a greater level of plasma membrane damage and cell cytotoxicity.

**Cell sorting.** Approximately 5 × 10^6^ cells were transfected with RNPs prepared using GFP-Cas9 and appropriate gRNA. After 24 h, the GFP^+^ cells were sorted using a BD FACS Aria fusion flow cytometer. The sorted GFP^+^ cells were cultured for 10–15 passages to observe long-term effects.

**RNAscope:** RNAscope (an in situ RNA hybridization technique) was performed using an assay kit from ACD (Newark, CA, USA). Briefly, 5 × 10^5^ cells were mounted on cover glasses (8 mm in diameter, Fisher Scientific) and cultured in a 24-well plate overnight, followed by fixation with 10% NBF, dehydration, and immobilization. Next, the slides were incubated with RNAscope hydrogen peroxide at room temperature (RT) for 10 min, followed by incubating with RNAscope Protease III (diluted 1:5 in 1X PBS) at RT for 10 min. Then, the RNAscope probe (V-HIV1-CLADEB-VIF-VPR-TAT-REV-VPU-ENV-NEF-TAR (Cat# 444061, ACD), which binds to the region 5042–9673 and is specific to HIV-1_NL4-3_ genome sequence (PubMed Accession no. AF324493.2), was hybridized at 40 °C for 2 h in a hybridization oven, then processed with RNAscope 2.0 HD detection reagent. Finally, the slides were dried in a 60 °C oven, rinsed in pure xylene, and immediately covered with 1 drop of EcoMount (Fisher Scientific) and a coverslip (40 × 24 mm, Fisher Scientific). Images were obtained using an EVOS microscope (Life Technologies, Carlsbad, CA, USA) and analyzed by QuPath software and GraphPad Prism (Version 6.01).

**Western blotting.** Western blot was performed to measure the expression levels of several key proteins involved in cellular activation and DNA damage and repair mechanisms. As described above, cells were PMA-stimulated for 2 h, followed by RNP nucleofection. Whole-cell lysates were prepared using RIPA buffer (Boston BioProducts, Ashland, MA, USA) in the presence of protease inhibitors (Thermo Scientific, Rockford, IL, USA). Protein concentration was measured by the Pierce bicinchoninic acid (BCA) protein assay kit (Thermo Scientific). Proteins extracts were separated by SDS-PAGE, transferred to polyvinylidene difluoride membranes, and pre-blocked with 5% nonfat milk in 0.1% Tween 20 and Tris-buffered saline (TBS). The membranes were incubated overnight with the following primary antibodies: anti-PD-1, anti-AKT, anti-pAKT^Ser473^, anti-pPTEN^Ser380^, anti-pBAD^Ser112^, anti-DNA ligase 4, anti-Ku70, anti-Ku80, or anti-GAPDH antibody as a loading control (Cell Signaling Technology, Danvers, MA, USA). The anti-p24 antibody was received from NIH AIDS Reagent Program. After washing, the membranes were incubated with appropriate horseradish peroxide-conjugated secondary antibodies (Cell Signaling Technology) for 2 h at room temperature. Protein bands were detected using Amersham ECL Prime Western blotting detection reagent (GE Healthcare Bio-Sciences, Pittsburgh, PA, USA). The bands were captured and quantified using the ChemiDoc MP imaging system (Bio-Rad, Hercules, CA, USA).

**Statistical analysis.** All data were analyzed using Prism 7 software (Irvine, CA, USA) and are expressed as mean ± SE. Comparisons between two groups were made using a parametric paired or unpaired *t*-test for normally distributed data or non-parametric Wilcoxon paired *t*-test or Mann–Whitney U-test for non-normal distributions. Comparisons among multiple groups were made using a one-way ANOVA at a 95% confidence level (Tukey’s honest significance test). *p* values < 0.05 (*) were considered statistically significant and *p* values < 0.01 (**), <0.001 (***), or <0.0001 (****) were considered very significant.

## Figures and Tables

**Table 1:** The primers used in the T7E1 mismatch cleavage assay.

Primers	Forward (5′-3′)	Reverse (5′-3′)
gRNA-3 flanking	AAGGGAAGGCCAGGGAATTTTCTTCAGAGC	TTTTATTTTTTCTTCTGTCAATGGCC
gRNA-4 flanking	TAGCAGCATTAATAAAACCAAAACAGATAAAGCC	ATGAGCTCTTCGTCGCTGTCTCCGC

**Table 2:** The primers used for target site sequencing.

Primers	Forward (5′-3′)	Reverse (5′-3′)
gRNA-3 sequencing	AATTAAAGGAAGCTCTATTAGATACAGG	TATTGTATGGATTTTCAGGCCC
gRNA-4 sequencing	TTGGGCAGGAGTGGAAGCC	ATGAGCTCTTCGTCGCTGTCTCCGC

## Data Availability

Not applicable.

## Supplementary Materials

The following supporting information can be downloaded at: https://www.mdpi.com/article/10.3390/v14091902/s1, Figure S1: Sequence conservation of gRNA-3 and gRN-4; Figure S2: Transfection efficiency and cell sorting of J1.1 cells transfected with GFP-labeled Cas9 RNP; Figure S3: DNA sequencing reveals the cleavage site of gRNA-4; Figure S4: DNA sequencing confirms the cleavage site by gRNA-3; Table S1: Features of gRNAs used in this study.
