# Supplementary material for: Synthetic gRNA/Cas9 Ribonucleoprotein Inhibits HIV Reactivation and Replication
Source: Viruses. 2022 Aug 28;14(9):1902. doi: 10.3390/v14091902 (PMC9500661; doi:10.3390/v14091902)
Supplement: Supplementary file 1 [file viruses-14-01902-s001.zip › viruses-1843125-SM.pdf]

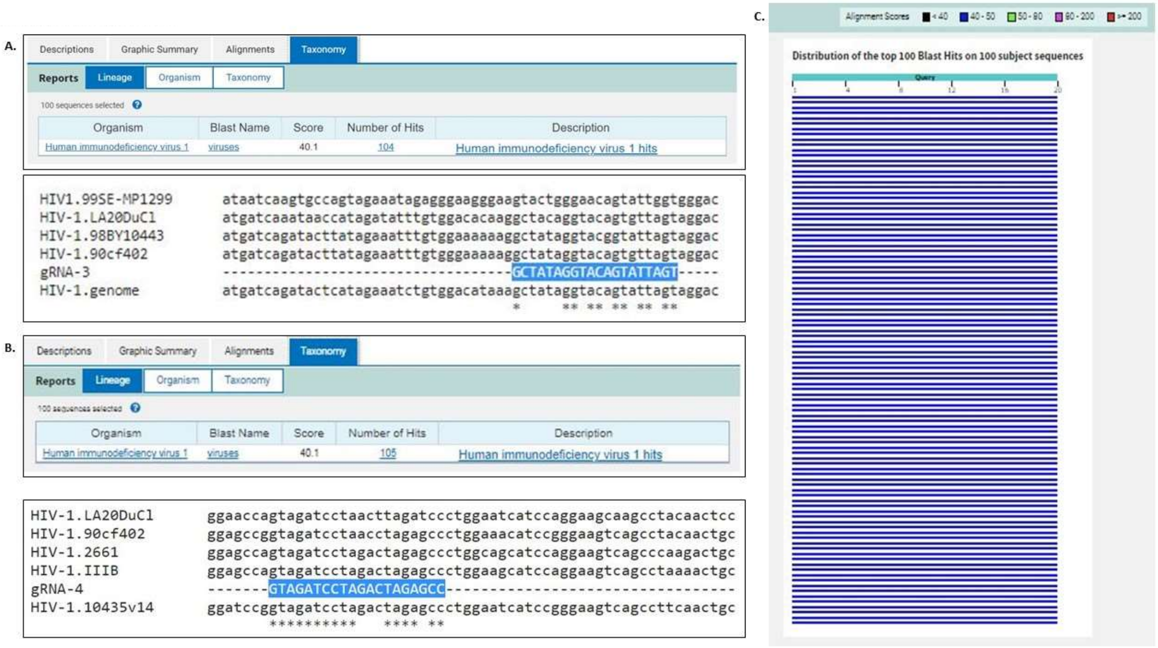


**Fig.S1. Sequence conservation of gRNA-3 and gRN-4. A)** NCBI nucleotide blast revealed a total of 104 hits with gRNA-3 (highlighted in blue) across the published HIV-1 genome sequences (upper panel). A comparison of gRNA-3 sequence conservation across different HIV-1 strains using Clustal Omega multiple sequence alignment tool (lower panel). The HIV-1 strains used are 99SE-MP1299 (Senegal), LA20DuCI (Romania), 98BY10443 (Belarus), and 90cf402 (Central Africa). The HIV-1 complete genome is based on NCBI database. **B)** NCBI nucleotide blast revealed a total of 105 gRNA-4 (highlighted in blue) targets across the published HIV-1 genome sequences (upper panel). The gRNA-4 sequence conservation was compared within different HIV-1 strains using Clustal Omega multiple sequence alignment tool (lower panel). The HIV-1 strains used are LA20DuCI (Romania), 90cf402 (Central Africa), 2661 (United States of America), IIIB (United Kingdom), and 10435v14 (Uganda), based on NCBI database. **C)** Alignment score of the gRNAs across the published sequence revealed a uniform alignment with the first 100 sequences with a score of 40.1.

This score is dependent on factors such as mismatches, gaps, sequence length, and identical residues.

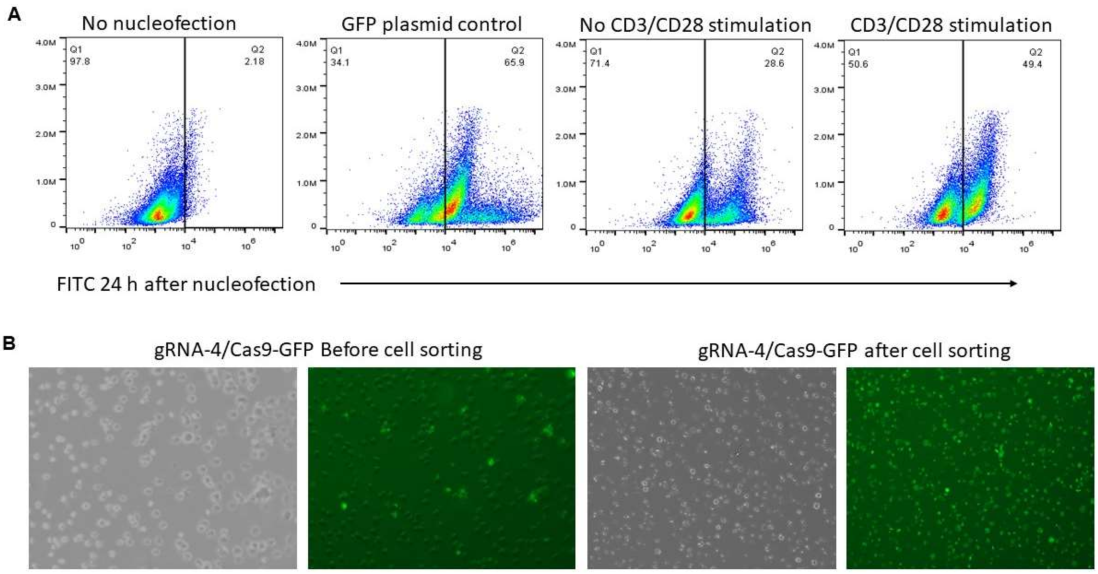


**Fig.S2. Transfection efficiency and cell sorting of J1.1 cells transfected with GFP-labeled Cas9 RNP. A)** Transfection efficiency of GFP plasmid control or GFP-labeled Cas9 RNP in J1.1 cells with or without anti-CD3/CD28 stimulation was analyzed by flow cytometry. **B)** J1.1 cells were transfected with gRNA4/Cas9-GFP, followed by cell sorting of $GFP^+$ cells and fluorescence microscopy analysis.

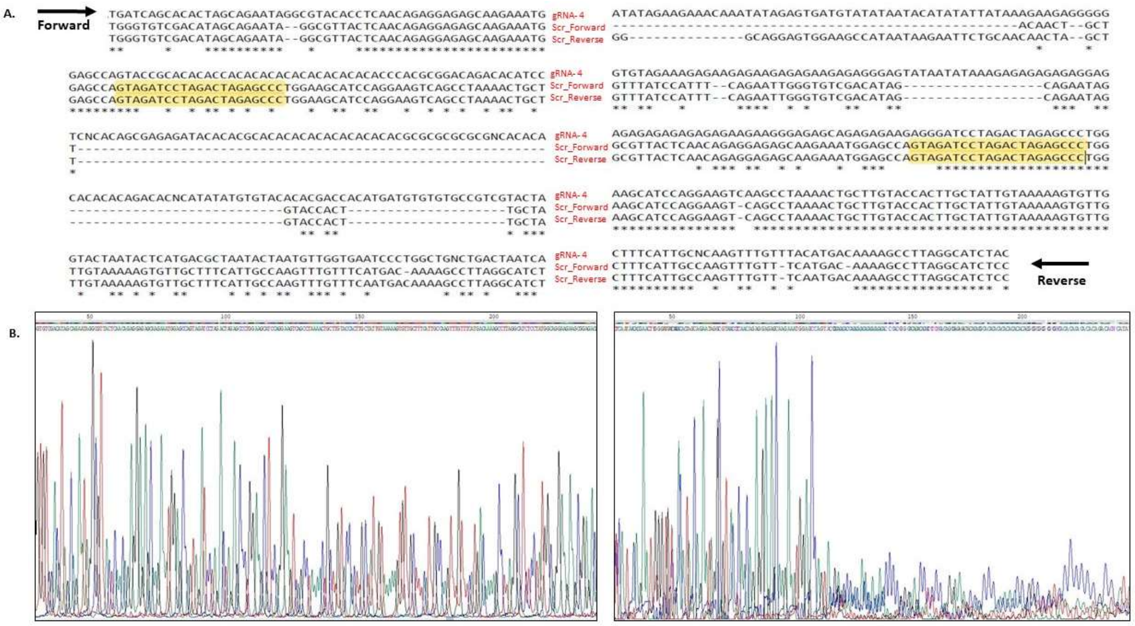


**Fig.S3. DNA sequencing reveals the cleavage site of gRNA-4. A)** Sanger sequencing data of gRNA-4 cleavage site (highlighted in yellow) compared to the scramble sequence, followed by PCR to amplify ~200 bases away from the cleavage site (see Table 2)**.** Both forward and reverse primers were used for sequencing. **B)** Nucleotide peaks were observed in the scramble control sequence (left) and the gRNA-4 cleaved site (right). The color code for the nucleotide bases (A- Green, T- Red, C- Blue and G- Black) match the color of the peaks.

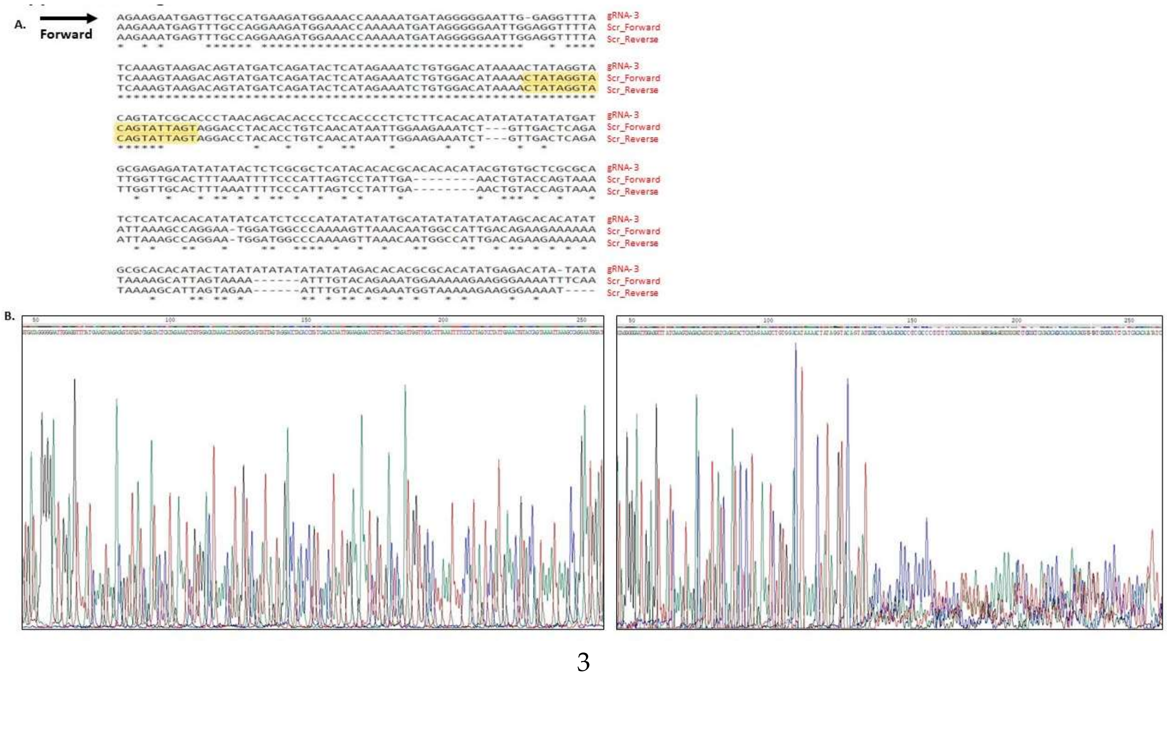

**Fig.S4. DNA sequencing confirms the cleavage site by gRNA-3. A)** Sanger sequencing data of gRNA-3 cleavage site (highlighted in yellow) compared to the scramble sequence, followed by PCR to amplify ~200 bases away from the cleavage site (see Table 2**).** Forward primer was used for sequencing. **B)** Nucleotide peaks were observed in the scramble control sequence (left) and the gRNA-3 cleaved site (right). The color code for the nucleotide bases (A- Green, T- Red, C- Blue and G- Black) match the color of the peaks.

| Name | gRNA3 | gRNA4 | gRNA5 | gRNA6 | gRNA7 | gRNA8 | gRNA9 | gRNA10 |
|---|---|---|---|---|---|---|---|---|
| Length | 23 | 23 | 23 | 23 | 23 | 23 | 23 | 23 |
| Start | 2462 | 5835 | 9102 | 77 | 2220 | 4593 | 7812 | 8447 |
| End | 2485 | 5858 | 9125 | 100 | 2243 | 4616 | 7835 | 8470 |
| Strand | plus | minus | plus | plus | minus | minus | plus | minus |
| Nucleotide sequence | GCTATAGGTACAGT ATTAGT NGG | GGCTCTAGTCTAGGA TCTAC NGG | GACAAGATATCCTTGA TCTG NGG | GATTGGCAGAACTACA CACC NGG | GGATACAGTTCCTTG TCTAT NGG | GGCGGCCTTAACTG TAGTAC NGG | GTCAATGACGCTGA CGGTAC NGG | GGATCCGTTCACTA ATCGAA NGG |
| Gene Name | NA | NA | NA | NA | NA | NA | NA | NA |
| Transcripts | NA | NA | NA | NA | NA | NA | NA | NA |
| Transcript:: Exon | NA | NA | NA | NA | NA | NA | NA | NA |
| Number of Cpg Islands hit | NA | NA | NA | NA | NA | NA | NA | NA |
| Sequence around the cutside | NA | NA | NA | NA | NA | NA | NA | NA |
| %A, %C, %T, %G | 32, 12, 28, 28 | 28, 32, 20, 20 | 28, 16, 32, 24 | 32 28 12 28 | 32 24 24 20 | 24 32 20 24 | 24 28 16 32 | 24 24 32 20 |
| S-Score | 100 | 100 | 100 | 80 | 100 | 40 | 60 | 60 |
| A-Score | 0 | 0 | 0 | 0 | 0 | 0 | 0 | 0 |
| E-Score | 59.8476 | 51.9487 | 55.2932 | 59.4405 | 59.176 | 51.1169 | 56.4779 | 59.1206 |
| Percent of total transcripts hit | NA | NA | NA | NA | NA | NA | NA | NA |
| Target | * | * | * | * | * | * | * | * |
| Match-start | 0 | 0 | 0 | 0 | 0 | 0 | 0 | 0 |
| Match-end | 0 | 0 | 0 | 0 | 0 | 0 | 0 | 0 |
| Matchstring | NA | NA | NA | NA | NA | NA | NA | NA |
| Editdistance | 0 | 0 | 0 | 0 | 0 | 0 | 0 | 0 |
| Number of Hits | 1 | 1 | 1 | 2 | 1 | 4 | 3 | 3 |
| Direction | NA | NA | NA | NA | NA | NA | NA | NA |
| CDS_score | 0 | 0 | 0 | 0 | 0 | 0 | 0 | 0 |
| Exon_Score | 0 | 0 | 0 | 0 | 0 | 0 | 0 | 0 |
| Seed_GC | 0.3 | 0.5 | 0.4 | 0.5 | 0.4 | 0.4 | 0.6 | 0.3 |
| Doench_Score | 0.439160127 | 0.077144417 | 0.203672831 | 0.137563451 | 0.394009732 | 0.038300739 | 0.019639276 | 0.4385964 |
| Xu_score | 0.253221936 | 0.020294268 | 0.160990732 | 0.334462527 | 0.164793987 | 0.117548146 | 0.20425613 | 0.217434778 |
| Chromosome | NA | NA | NA | NA | NA | NA | NA | NA |
| Genomic start | 1962 | 5335 | 8602 | -423 | 1720 | 4093 | 7312 | 7947 |
| Genomic End | 1985 | 5358 | 8625 | -400 | 1743 | 4116 | 7335 | 7970 |

**Table S1. Features of gRNAs used in this study.** E-CRISP online tool was used to design the gRNAs with in-built parameter calculation. Characteristics of the selected gRNAs, including their name, lengths, start/end positions, plus/minus strands, nucleotide sequences, % of ATGC nucleotides, seed GC contents, S-/E-/Doench-scores, and number of hits, etc. are summarized.
